# Biomass Microcapsules with Stem Cell Encapsulation for Bone Repair

**DOI:** 10.1007/s40820-021-00747-8

**Published:** 2021-12-02

**Authors:** Lei Yang, Yuxiao Liu, Lingyu Sun, Cheng Zhao, Guopu Chen, Yuanjin Zhao

**Affiliations:** 1 Department of Rheumatology and Immunology, The Affiliated Drum Tower Hospital of Nanjing University Medical School, Nanjing, 210008 People’s Republic of China; 2 Wenzhou Institute, University of Chinese Academy of Sciences, Wenzhou, 325001 Zhejiang People’s Republic of China; 3 Oujiang Laboratory (Zhejiang Lab for Regenerative Medicine, Vision and Brain Health), Wenzhou, 325001 Zhejiang People’s Republic of China; 4 State Key Laboratory of Bioelectronics, School of Biological Science and Medical Engineering, Southeast University, Nanjing, 210096 People’s Republic of China

**Keywords:** Microcapsule, Bone repair, Stem cell therapy, Microfluidics, Electrospray

## Abstract

Bone defects caused by trauma, tumor, or osteoarthritis remain challenging due to the lack of effective treatments in clinic. Stem cell transplantation has emerged as an alternative approach for bone repair and attracted widespread attention owing to its excellent biological activities and therapy effect. The attempts to develop this therapeutic approach focus on the generation of effective cell delivery vehicles, since the shortcomings of direct injection of stem cells into target tissues. Here, we developed a novel core-shell microcapsule with a stem cell-laden core and a biomass shell by using all-aqueous phase microfluidic electrospray technology. The designed core-shell microcapsules showed a high cell viability during the culture procedure. In addition, the animal experiments exhibited that stem cell-laden core-shell microcapsules have good biocompatibility and therapeutic effect for bone defects. This study indicated that the core-shell biomass microcapsules generated by microfluidic electrospray have promising potential in tissue engineering and regenerative medicine.

**Supplementary Information:**

The online version contains supplementary material available at 10.1007/s40820-021-00747-8.

## Highlights

- A novel stem cell delivery core-shell biomass microcapsule was generated by an all-aqueous phase microfluidic electrospray technique.
- Microcapsule with a certain mechanical strength and porous structure is beneficial for substances exchange between cells and the external environment, together with avoiding cell damage during treatment.
- Core-shell microcapsule provided a favorable cell growth microenvironment, which mimics a physicochemical microenvironment and protects the cells from the immune attack from body.

**Supplementary Information:**

The online version contains supplementary material available at 10.1007/s40820-021-00747-8.

## Graphical abstract

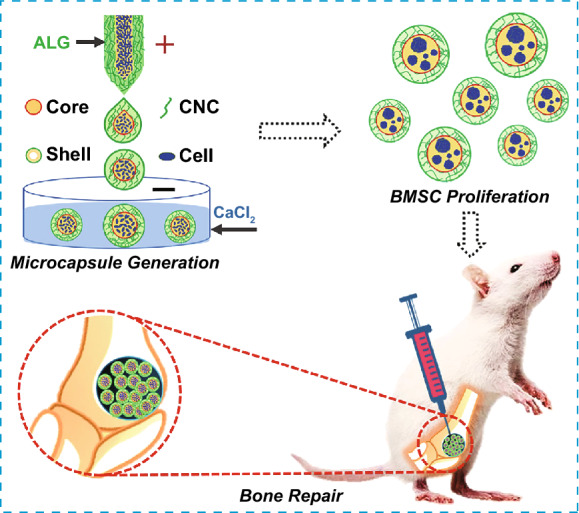

## Introduction

Diseased or damaged bone tissue caused by trauma, tumor, or osteoarthritis will lead to significant bone loss [1, 2]. In general, the supply of autografts, allografts, non-metallic and metallic implants is the common approaches for treating bone defects in clinic [3–5]. However, these strategies usually have intrinsic drawbacks, such as the inadequate donors, inflammatory responses, and integration failure [4, 5]. In order to overcome these limitations, bone tissue engineering has been proposed. Stem cells, as an essential part of tissue engineering, have attracted increasing attention due to their strong self-renewal capacity, multilineage differentiation, paracrine effects, anti-inflammatory capacity, and immunomodulatory ability [6, 7]. Although stem cell therapy has become a promising clinical approach [8], direct injection of stem cells into the target tissues will possibly result in decreasing cell viability, retention, and engraftment, which have largely limited the practical application of stem cell therapy [6]. Instead, the development of designed cell delivery systems has been expected to overcome the above-mentioned limitations. Generally, numerous synthetic or natural biomaterials have been used to construct cell delivery vehicles, such as hyaluronic acid (HA) [9–11], gelatin [12, 13], gelatin methacryloyl (GelMA) [14–16], and poly (N-isopropylacrylamide) (pNIPAM) [17, 18]. Despite many advantages and achievements, these materials are limited and expensive due to the complex processing environment, resource constraints, and poor mechanical property. In contrast, biomass, the reproducible organic materials derived from plants and animals, is fast-growing, abundant, and low-cost. Thus, the development of biomass-based cell delivery vehicles with excellent biocompatibility and favorable mechanical properties for bone defect treatments is still anticipated.

**Fig. 1:**
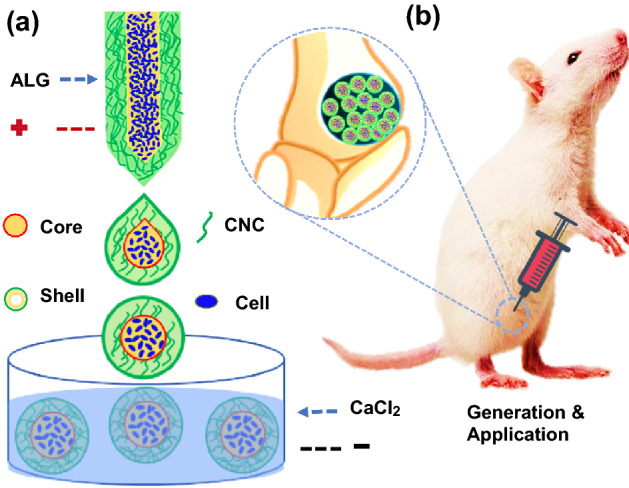
Schematic illustrations of the generation and application of the stem cell-laden microcapsules. **a** The microcapsules generation process by microfluidic electrospray. **b** The stem cell-laden core-shell microcapsules for bone repair

In this paper, we develop a novel stem cell delivery microcapsule with biomass shell (cellulose nanocrystals (CNC) and alginate (ALG)) by using all-aqueous phase microfluidic electrospray technique for bone repair, as schemed in Fig. 1. CNC, which is extracted from cellulose (the most extensive biomass material on land), have demonstrated great application values in biomedical engineering including drug delivery [19], biosensors [20], and tissue engineering [21], because of its renewability, degradability, biocompatibility, and excellent mechanical performance. ALG, separating from the most abundant algal biomass in the water, has found a wide range of applications in many fields, such as drug release [22, 23], cell delivery [23], and tissue engineering [24], owing to its biocompatibility, low cost, and easy gelation. Various microfluidic platforms, such as capillary microfluidic [25], centrifugal microfluidic [26], and non-planar microfluidic [27, 28], can precisely control the monodispersity, size, compartmentalization, and structure of microparticles or microcapsules [29–31], which have been widely used in chemical analysis [32], drug delivery [33, 34], medical diagnosis [35] and cell encapsulation [26, 30]. Nevertheless, despite the merits, the common microfluidic device often uses organic solvents as a continuous phase to produce droplets, which may cause a debatable biocompatibility, and brings about bacterial pollution, decreases productivity, and increases production costs in the following process of removing organic reagents. Besides, some traditional microfluidic methods require precision experimental equipment and trained laboratory staff, which limits the wide application of microfluidic technology. Thus, how to construct suitable cell delivery vehicles through more appropriate approaches and simpler devices is still worth pondering.

For this purpose, a simpler microfluidic electrospray device with double coaxial capillaries was fabricated for rapidly generating uniform core-shell microcapsules with a cell-containing liquid core and a mixed biomass (CNC and ALG) shell. The designed microcapsules could be generated by one-step method via adjusting the tension between internal and external phase, which avoid the problems of biological toxicity from organic solvents, bacterial pollution, high cost, and low productivity. Meanwhile, the shell of microcapsules with CNC provided a certain mechanical strength and the characteristic porous structure, which was beneficial for substances exchange between cells and the external environment, as well as avoiding mechanical damage during treatment. In addition, the generated core-shell structures could provide a favorable cell growth microenvironment, which was able to better mimic the real physicochemical microenvironment in vivo and protect the cells from the immune attack of body. These features make the designed core-shell microcapsules ideal for cell encapsulation and cell delivery, and thus promising in stem cell-based bone repair.

## Experimental Section

### Materials

Trypsin, CaCl_2_, DMSO, and proteinase K solution were bought from Sigma (St. Louis, MO, the USA). CMC and high viscosity ALG were bought from Aladdin (Shanghai, China). CNC were achieved from Beike 2D materials Co., Ltd (Beijing, China). BMSC of rat was purchased from Cyagen Biosciences (Guangzhou, China). Preosteoblast cell line MC3T3-E1 was obtained from the Cell Bank of Chinese Academy of Sciences (Shanghai, China). DMEM cell culture medium was obtained from Hy Clone (Logan, Utah, the USA). Fetal bovine serum (FBS) was purchased from Gibco (Grand Island, NY, the USA). Picogreen DNA quantification assay, blue fluorometric dsDNA quantitation kit, and Calcein AM were purchased from Invitrogen (Shanghai, China). The antibodies of OPN and OCN were obtained from Abcam Biotechnology (Shanghai, China). MTT was bought from Biyuntian (Shanghai, China). PBS was prepared in laboratory. The 10- to 12-week-old male SD rats were supplied by Comparative Medicine of Jinling Hospital (Nanjing, China).

### Production of Microfluidic Device

The coaxial electrospraying nozzle device was constructed by coaxially assembling two round capillaries on a glass slide which served as a plate (Fig. S1). The diameters of the inner and outer capillary were 100 and 300 μm, respectively. The spindle inner capillary was then coaxially inserted into the outer capillary. Then, connection points of the devices were sealed with dispensing needles and transparent epoxy resin (Devcon 5 Minute Epoxy).

### Cell Culture

BMSC and MC3T3-E1 were cultured in DMEM with 10% FBS in a humidified atmosphere of 5% CO_2_ at 37 °C. The culture medium was changed every 3 days. When the cells reached 80–90% intensity, they were digested with 0.25% w/v trypsin for 3 min. Then, the cells were centrifuged at 1000 rpm for 3 min and resuspended in sodium carboxymethylcellulose solution for further use. The stem cell-laden microcapsules were collected from calcium chloride solution and washed with DMEM for 3 times and then cultured for different days.

### Generation of Cell-Laden Microcapsules

Before the experiment, the microfluidic device was sterilized with 75% alcohol and UV irradiation for 20 min. The microfluidic device consisted of four units including fluid inlets (micro-injection pump), droplet generation (coaxial nozzle device), electric field (high voltage power), and microcapsule fabrication (CaCl_2_ solution). In a typical experiment, 2% biomass (ALG/CNC) in a ratio of 3 to 1 (w/w)) was used as the shell solution, while DMEM culture media containing with 1 × 10^8^/mL passage 3–4 BMSC and 2% CMC was employed as the core solution. The flow rates of the outer (shell) phase and inner (core) phase were 100 µL and 300 µL min^−1^, respectively. The outer sodium biomass shell of capsules was crosslinked by 2% CaCl_2_, forming the final cell-laden hydrogel microcapsules. The crosslinking time was reduced to 5 min, and then, the cell-laden microcapsules were quickly washed with culture medium to remove the residual CaCl_2_. At last, the cell-laden microcapsules were cultured in DMEM culture media and stained by Calcein AM for observing the cell morphology and proliferation.

### Characterization

The pictures of the microcapsules with or without cells were captured by a confocal laser scanning microscope (Zeiss LSM700, 1–7 day) and an inverted fluorescence microscope (OLYMPUS BX51, 7–28 day). The microstructures of the microcapsules were characterized by a scanning electron microscope (Hitachi, S300N). The diameters of particles were calculated by software AOS Imaging Studio V3.4.2.

### Cell Proliferation

After culturing for different times, the proliferation of BMSC in different doses of biomass microcapsules was determined by using a blue fluorometric dsDNA quantitation kit. Simply, the BMSC-laden microcapsules were lysed in after two freeze–thaw cycles at -80 °C. Then, the microgels were ground with a pestle to release cellular DNA and centrifuged at 12,000 rpm for 10 min. After centrifugation, the supernatants were collected and added with DNA-conjugated dye. The DNA amount in each culture was calculated based on a standard curve. To detect the biocompatibility, BMSC and MC3T3-E1 cells were divided into different groups including the Control, Capsule, Capsule-BMSC and tested by MTT and Calcein AM staining. When the cells were cultured for 0–3 days, they were treated with 0.3 mg mL^−l^ MTT for 3 h. Then, medium was replaced with DMSO for 20 min. At last, the supernatant was collected for detecting the OD value at 570 nm. At the same time, the cells were stained by 10 µM Calcein AM staining for cell morphology observation.

### Osteogenic Induction

The generated biomass microcapsules with BMSC were cultured in DMEM culture media for 1 week to induce cell aggregation and proliferation. Then, the microcapsules were then divided into two groups: control and osteogenic induction group. The control groups continued to use the DMEM medium for cell culture and osteogenic induction group was cultured in the DMEM medium containing 10 mM β-sodium glycerophosphate, 100 mM dexamethasone, and 50 μg mL^−1^ vitamin C for another 21 days’ culture. At last, the microcapsules were collected for the evaluation of the osteogenic differentiation by alizarin red staining and osteogenic gene expression including Runx2, Ocn, and Opn. The detailed primer sequences are listed in Table S1.

### Rat Model Establishment

Animal experiments were approved by the Animal Investigation Ethics Committee of The Affiliated Drum Tower Hospital of Nanjing University Medical School. The 10–12 weeks old SD rats were general anesthesia via intraperitoneal injections of 10% chloral hydrate at 0.5 ml/100 g, and then, rats were randomly divided into four groups, including control, BMSC, Capsule, and Capsule-BMSC groups. The bone defect model was done as previous studies [2, 4]. In brief, the distal femurs were pierced with an electric drill to make a bone defect with diameter 2 mm and depth 3 mm. Then, the sterilized Capsule, BMSC and Capsule-BMSC combined with 0.3 mL physiological saline solution were injected into the defects. After treatment for 8 weeks, the rats were sacrificed, and the bone was fixed in 4% paraformaldehyde for micro-CT and histological analysis. The 3D reconstruction data from micro-CT were further used for BMD and BV/TV quantitative analyses. Then, the samples were decalcified in 12% EDTA, dehydrated in ethanol, and embedded in paraffin. At last, the embedded bone in paraffin was stained with H&E and the antibody of OPN (1:200 dilutions) and OCN (1:150 dilutions) after paraffin section treatment. At the same time, the main organs from the microcapsule groups were collected for HE staining. Besides, the carbon black ink labeled microcapsules were used to observe the distribution of microspheres in bone defects area.

## Results and Discussion

### Generation of the Stem Cell Encapsulated Core-shell Microcapsules

**Fig. 2:**
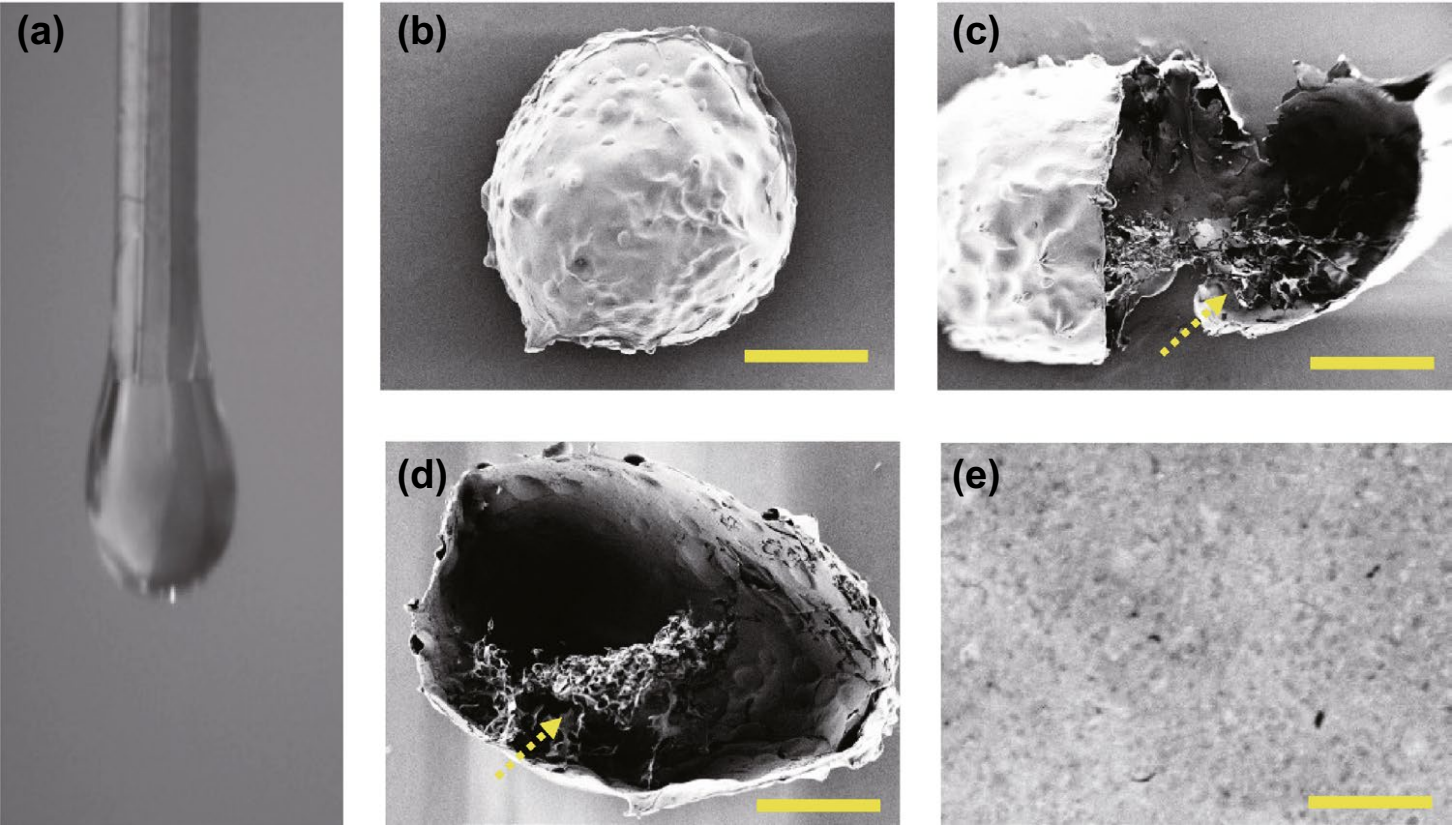
The preparation of the stem cell-laden core-shell microcapsules. **a** The real-time image of the microfluidic electrospray process of the microcapsules. **b**–**e** Scanning electron microscope images of the whole microcapsule (**b**), the interior of stem cell-encapsulated microcapsules (**c**), stem cells inside the microcapsules (**d**), and the enlarged view of the surface of microcapsules (**e**). The yellow arrow indicates the cells in the microcapsules. Scale bar in (**b**, **c**, and **d**) is 100 μm and in (**e**) is 10 μm

**Fig. 3:**
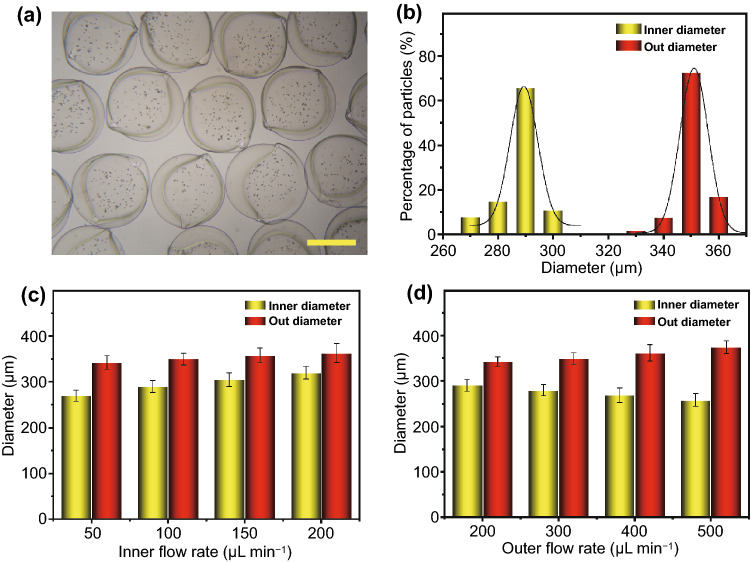
Factors controlling the microcapsules size. **a**, **b** The morphology (**a**) and size distribution (**b**) of produced microcapsules. **c**, **d** The relationship of inner (**c**) and outer (**d**) phase flow rates on microcapsules size. The scale bar is 200 μm

The schematic overview of the experiment is shown in Fig. 1. Stem cell (bone marrow mesenchymal stem cell, BMSC) encapsulated microcapsules were generated by a double-emulsion capillary microfluidic electrospray device as shown in Fig. 1a. The image of the tube-in-tube device is given in Fig. S1a. The microfluidic device has two aqueous phases for making the core and shell of the microdroplets. The inner phase was the mixture of stem cells and culture medium containing 2% carboxymethylcellulose (CMC), and the outer phase is the 2% biomass solution (ALG and CNC in a ratio 3:1). Both of inner phase and outer phase were pumped into the device through syringe pumps. The inner solutions could form laminar flows and were sheathed by the outer biomass solutions at the merging point of these fluids (Fig. S1b). Then, the co-flow was broken up into the droplets by the outer electric field (Fig. 2a). The generated microcapsules were collected by a gelling pool containing 2% calcium chloride (CaCl_2_) solution where the biomass layer of the capsules could be rapidly crosslinked (Fig. 1a). After the crosslinking process, cell encapsulated core-shell structures microcapsules were quickly collected and moved into culture medium. The stem cells encapsulated microcapsules were also characterized by the optical microscope (Fig. 3a) and scanning electron microscope (Fig. 2b-e). The microcapsules had spherical morphology with a rough surface and small voids due to the adding of CNC (Fig. 2a, e). In contrast, microcapsules with shells generated only from ALG was easy to collapse and had no voids (Fig. S2a, b). Because ALG was composed of α-l-guluronic acid (G) units, which formed hydrogel by crosslinking of the G residues with the presence of Ca^2+^ [36]. When the biomass solution containing CNC, G residues of ALG can induce a higher degree of coordination with Ca^2+^ [37]. In order to ensure the activity of encapsulated cells, the collection process was usually finished in 5 min, and the collected microcapsules were washed with culture medium for 3 times to remove un-crosslinked calcium ions, since high dose of Ca^2+^ usually triggers cell apoptosis. Moreover, the phosphate-buffered saline (PBS) was not suitable for washing the microcapsules, because it would cause the microcapsules to swell. The possible mechanism of this phenomenon could be ascribed to that the monovalent cation (k^+^ and Na^+^) in PBS solution replaced the calcium ions in the crosslinked ALG and CNC. In addition, the cell-laden core-shell structured microcarriers are widely used in tissue engineering. However, most of the microcarriers or core-shell structured capsules were generally produced by organic solvent, which would cause cell death and protein denaturation. In this study, we adjusted the viscosity of internal phase liquid by adding CMC to form the core-shell structures at the water–water interface and regulated voltage strength to control the size of the microcapsules. As CMC is a natural, biocompatible, and biodegradable anionic polymer that has been widely used in tissue engineering, it can be helpful for cell adherence and proliferation [38].

### Factors Controlling the Microcapsules Size

In a typical experiment, flow rates of the inner phase and outer phase were 100 and 300 μL min^−1^, and the diameter of inner capillary and outer capillary was 100 and 300 μm, respectively. The strength of the electric field is 8 kv, and the collecting distance is 5 cm. In this system, the generated microcapsules showed great uniformity, and the size of microcapsules (diameter: 351.1 ± 16.3 μm) or their cores (diameter: 293 ± 12.8 μm) conformed to the standard size distribution (Fig. 3a, b). We first studied the factors that affecting the size of microcapsules, including impulse electric field voltage, collecting distance and concentration of the biomass solution. We found that the concentration of the biomass and collecting distance was positively correlated with the diameter of microcapsules (Fig. S3a, b). In addition, the diameter of the microcapsules began to decrease with the increasing voltage (Fig. S3c), which was consistent with previous studies [39, 40]. The reason could be ascribed to that high dose of the biomass solution demonstrated a higher adhesion force between microcapsules, and a larger diameter of outer capillary led to larger droplets and therefore larger microcapsules [26]. In addition, the larger the collection distance was, and the larger the microcapsules were. We further detected the relationship between flow rates of inner and outer phases and the diameters of core-shell capsules (Fig. 3c, d). The results showed that the increasing flow rate of inner phase led to the increasing diameter of cores and the decreasing shell thickness, as well as the slightly increasing diameter of the entire capsule (Figs. 3c and S4). At the same time, the increasing flow rate of outer phase could enlarge the diameter of microcapsules and the shell thickness, while decrease the core diameter (Fig. 3d).

In general, the microcapsules diameter was controlled by the common action of electric field force and liquid surface tension at the nozzle [39, 40]. Taylor cone appears at the nozzle when the electric field force gradually increases. When the electric field action is higher than the liquid surface tension, the droplets will break through the restraint at the nozzle and squirt from the tip of Taylor cone. Therefore, the size of microcapsules also depends on the strength of the electric field and the surface tension of the droplets [41]. With the increase in capillary diameter and biomass dose, the surface tension increased, which caused the microcapsules diameter increased. In addition, too large collection distance decreased the surface charge of liquid, so the microcapsules diameter increased [34, 39]. Moreover, the shell thickness depended on the relative velocity of the internal and external phases. The increasing inner flow rate decreased the shell thickness, while increasing outflow rate could increase the shell thickness. Furthermore, for high concentration of biomass, higher voltage should be applied; otherwise, the microcapsules would get bigger. Unfortunately, high voltage would damage the cell membrane and thus lead to cell death.

### Cell Proliferation and Biocompatibility Test

**Fig. 4:**
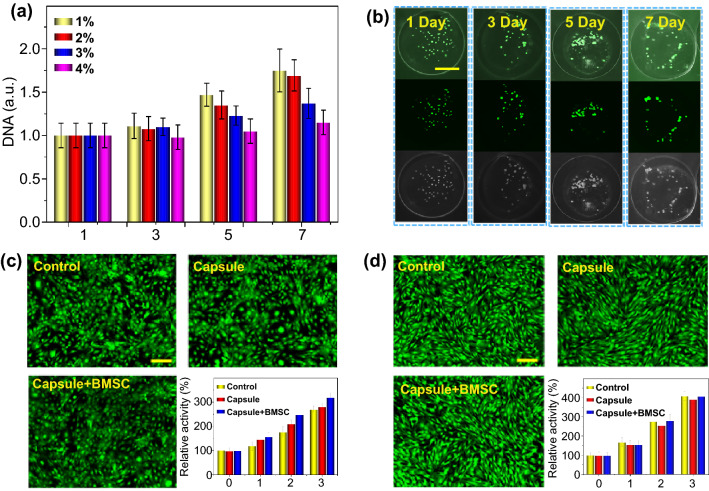
Cell proliferation and biocompatibility test in vitro*.* **a** The cell proliferation in different concentrations of biomass microcapsules. **b** The morphological changes of cells after 1–7 days culture in microcapsules, respectively. **c** Biocompatibility test when co-cultured with MC3T3-E1 after 3 days. **d** Biocompatibility test when co-cultured with BMSC after 3 days. Scale bar in (**b**) is 100 μm and in (**c** and **d**) is 50 μm

The biomass microcapsules allowed a rapid cell growth during 1–7 days culture (Fig. 4). We also detected the cell proliferation in microcapsules with different concentrations of biomass. When the concentrations of biomass were increased, the amount of DNA of BMSCs in the microcapsules decreased, which indicated that the cell proliferation was decreased (Fig. 4a) because the high dose of hydrogel hindered the nutrients to enter the core from the culture medium. While hydrogel with too low concentrations would lead to a poor mechanical strength, which caused mechanical damage of cells during the treatment. Hence, the 2% biomass was chosen for the following experiments based on the balance between mechanical strength and cell proliferation. At the same time, we detected the morphological changes of stem cells in the core-shell structured microcapsules through a confocal laser scanning microscope (Fig. 4b) and an inverted fluorescence microscope (Fig. S5). The results showed that the cells were dispersed at the start, and then, the cells clustered into cell aggregates with the extension of culture time. This could be ascribed to the bio-functionalization of microcapsules and chemical group of hydrogels, which contributed to cell growth, adhesion, and migration to the hydrogel microcapsules [42]. Osteoblasts are the main somatic cell of bone tissue, and BMSC is the main stem cell of bone tissue. Hence, the preosteoblast cell line MC3T3-E1 (Fig. 4c) and primary stem cell BMSC (Fig. 4d) were used for biocompatibility test on osteoblast and stem cell in vitro. The results showed that the preosteoblast and stem cell were increased after culturing for 3 days, and no obvious difference was observed in different groups including control group, Capsule group and Capsule-BMSC group (Fig. 4c, d). At the same time, the toxicity of microcapsules on different organs was investigated by hematoxylin–eosin (H&E) staining. The results indicated that there was no obvious abnormality in the main organs including heart, liver, spleen, lung, and kidney (Fig. S6). These data indicated that the hydrogel microcapsules have an excellent biocompatibility in vivo and in vitro. We also evaluated osteogenic differentiation ability of BMSC in the microcapsules by alizarin red staining and osteogenic-related genes analysis (Fig. S7). The alizarin red staining results indicated that calcium deposition of induction group was higher than the control group after 21 days treatment (Fig. S7a-c). Since the runt-related transcription factor 2 (Runx2), osteocalcin (OCN), and osteopontin (OPN) are key transcription factors involved in osteogenesis differentiation [43]. Hence, we detected these gene expressions after induction in vitro. The results showed that the osteogenic genes including Runx2, Ocn, and Opn obviously increased in the induction group than the control group (Fig. 7d, e). Taken together, all these data indicated the microcapsules can support the BMSC growth and osteogenic differentiation in vitro.

### Morphological Analysis of Bone Repair

**Fig. 5:**
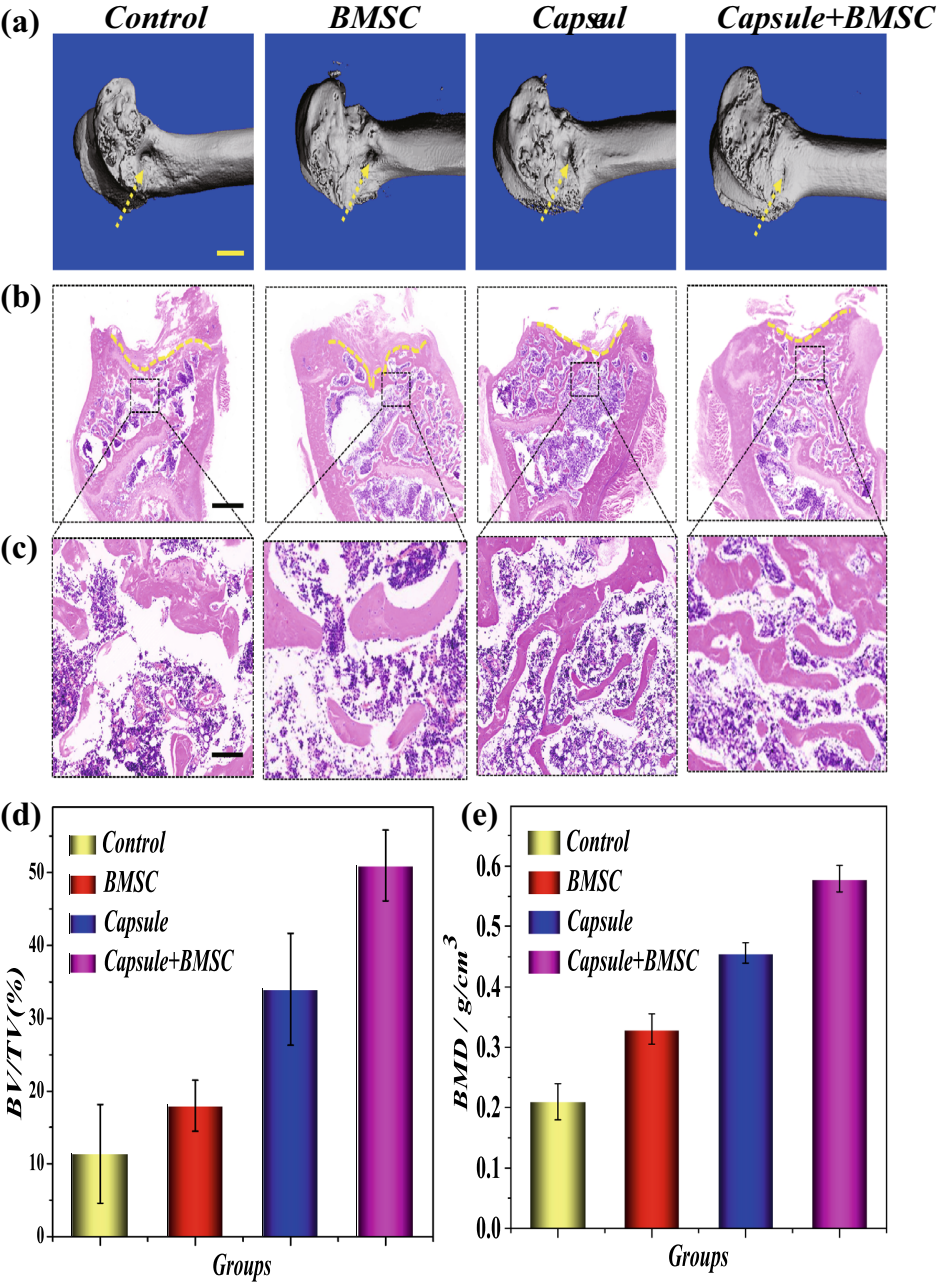
Representative **a** micro-CT reconstruction images, **b**, **c** H&E staining and **d**, **e** quantitative statistic of BV/TV and BMD of different groups: Control, Capsule, and Capsule-BMSC groups. Scale bars are 5 mm in (**a**), 1 mm (**b**) and 200 μm in (**c**). BV/TV: bone volume/total volume; BMD: bone mineral density

Before animal experiments, stem cells release ability of microcapsules were investigated in vitro*.* After incubation in physiological saline solution 4 weeks, the BMSC was completely released from the microcapsules (Figs. S8 and S9). To investigate the biodistribution of microcapsules in operation-induced bone defects model in vivo, the bone defects rats were treated with the carbon black ink labeled microcapsules. After 2 weeks treatment, the carbon black ink labeled microcapsules were still stayed in the bone defect site (Fig. S10). At the same time, the bone defects Sprague–Dawley rats with bone defects randomly divided into four groups including control, BMSC, Capsule, and Capsule-BMSC groups. After treating for 8 weeks, micro-computed tomography (micro-CT) and H&E staining were used to evaluate their treatment effects. The micro-CT shows that Capsules-BMSC treatment group significantly promoted bone regeneration compared to the un-loaded Capsules and control group. Besides, BMSC and Capsules treatment also accelerates bone repair (Fig. 5a, c). Meanwhile, H&E staining results showed that Capsule-BMSC treatment had a larger new bone volume and area, increased density of bone trabeculae, and accelerated bone regeneration (Fig. 5b, c). Besides, the bone mineral density (BMD) in BMSC, Capsules, and Capsule-BMSC group was higher than the control group (Fig. 5e). These results demonstrated that only BMSC and microspheres have a baseline osteogenic effect, and microspheres with BMSC have the best bone regeneration ability. The baseline osteogenic effect of microspheres may be attributed to natural bioactivity and the biocompatible scaffold of the microspheres, which is beneficial to the migration and proliferation of endogenous stem cells [44]. Besides, the release of Ca^2+^ from the microcapsules could induce osteogenic differentiation of endogenous stem cells via calcium/calmodulin signaling pathway [45–47].

### Osteogenic Protein Expressions

**Fig. 6:**
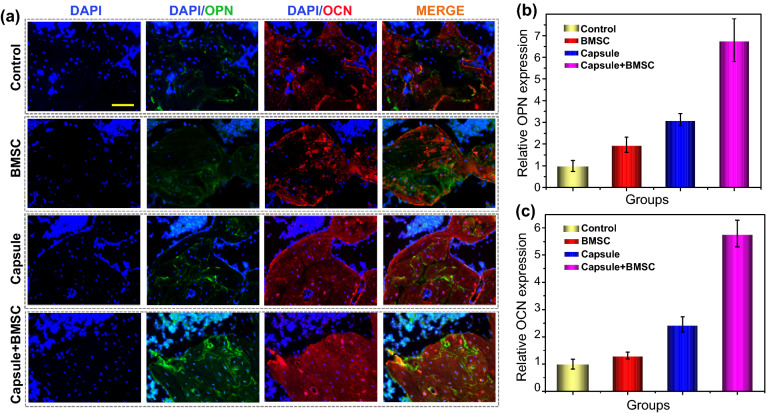
Osteogenic protein expressions in different groups. **a** Representative OPN and OCN staining in different groups. **b** The relative OPN expression in different groups. **c** The relative OCN expression in different groups. The scale bar is 50 μm. OCN: osteocalcin; OPN: osteopontin

In order to further characterize the bone regeneration process, we detected the expression level of OPN and OCN in different groups by immunofluorescence (Fig. 6). OPN promotes osteogenesis during bone regeneration, and OCN plays a key role in bone calcium metabolism [2]. The immunofluorescence results showed that the fluorescence intensity of OPN (Green) and OCN (Red) in the Capsule-BMSC groups was significantly higher than the control, BMSC and Capsule groups, indicating that Capsule-BMSC could effectively accelerated bone regeneration by promoting the OCN and OPN expressions (Fig. 6a-c). Besides, the expression of OPN and OCN was obviously higher in Capsule and BMSC groups compared to the control group, indicating that BMSC and biomass microcapsules have baseline osteogenic effect (Fig. 6a-c). These results indicated that BMSC, Capsule and Capsule-BMSC can promote bone repair by increasing the expressions of OPN and OCN, which was in accordance with the micro-CT and H&E results. These data demonstrated that the BMSC-encapsulated microcapsules could be applied for bone regeneration, and the possible reasons could be ascribed to that interstitial stem cells could secrete cytokines and some exosomes that stimulated OPN and OCN expression, thus accelerating bone regeneration [48–50]. Besides, the BMSC can migrate to damaged areas and differentiate into osteoblast. Only capsule treatment could also enhance the bone reconstruction to some extent compared with the control group, because the microcapsule provided a biocompatible scaffold which was helpful for the migration and proliferation of endogenous cells in the injuries portion. Besides, the release Ca^2+^ from the degradation of microspheres induced the endogenous stem cells differentiate into osteoblasts [45–47].

## Conclusions

We developed a kind of novel stem cell-encapsulated biomass microcapsules by microfluidic electrospray. The structure and size of the designed microcapsules could be controlled by the voltage, collection distance, and flow rates. The characteristic structure of the microcapsules provided a favorable cell growth microenvironment, mimicked the physicochemical microenvironment, and had satisfactory biocompatibility. Moreover, this biomass microcapsules culture system from microfluidic electrospray showed an excellent three-dimensional cell culture ability. Besides, the stem cell-loaded core-shell microcapsules could be used for the treatment of bone defect. It could be anticipated that the core-shell microcapsules are suitable for construction of organoids in vitro by utilizing different cell lines such as tumor cells. Based on these features, the designed core-shell microcapsules can be applied in many biomedical applications, such as cell encapsulation, developmental biology, pathology and drug screening, and relevant diseases treatment.

## Supplementary Information

Below is the link to the electronic supplementary material.

Supplementary file1 (PDF 775 kb)
