# Supplementary material for: Biomass Microcapsules with Stem Cell Encapsulation for Bone Repair
Source: Nanomicro Lett. 2021 Dec 2;14:4. doi: 10.1007/s40820-021-00747-8 (PMC8639896; doi:10.1007/s40820-021-00747-8)
Supplement: Supplementary file 1 — Supplementary file1 (PDF 775 kb) [file 40820_2021_747_MOESM1_ESM.pdf]

Supporting Information for

Biomass Microcapsules with Stem Cell Encapsulation for Bone Repair

Lei Yang[1, 2, 3], Yuxiao Liu[1, 4], Lingyu Sun[1, 4], Cheng Zhao[1], Guopu Chen[1], Yuanjin Zhao[1, 4, *]

[1] Department of Rheumatology and Immunology, The Affiliated Drum Tower Hospital of Nanjing University Medical School, 210008 Nanjing, P. R. China

[2] Wenzhou Institute, University of Chinese Academy of Sciences, Wenzhou, Zhejiang 325001, P. R. China

[3] Oujiang Laboratory (Zhejiang Lab for Regenerative Medicine, Vision and Brain Health), Wenzhou, Zhejiang 325001, P. R. China

[4] State Key Laboratory of Bioelectronics, School of Biological Science and Medical Engineering, Southeast University, Nanjing 210096, P. R. China

*Corresponding author. E-mail: yjzhao@seu.edu.cn (Yuanjin Zhao)

Supplementary Figures and Table

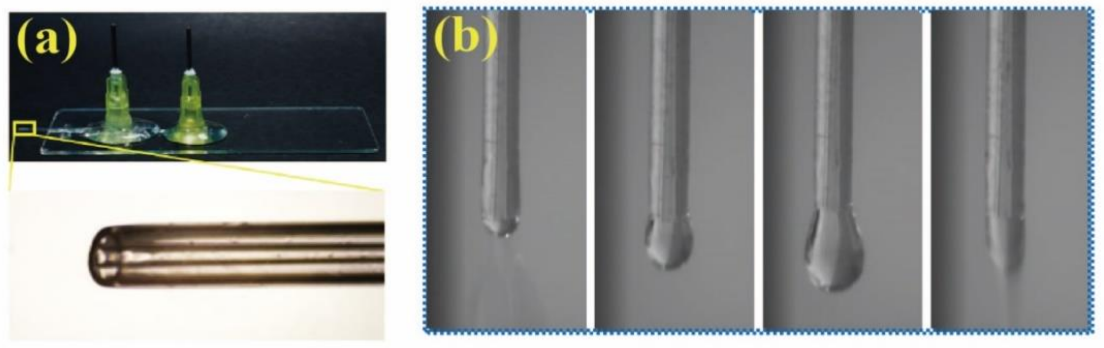

Fig. S1 Microcapsules generation device and process. **(a)** Microfluidic device. **(b)** Process of microcapsules generation

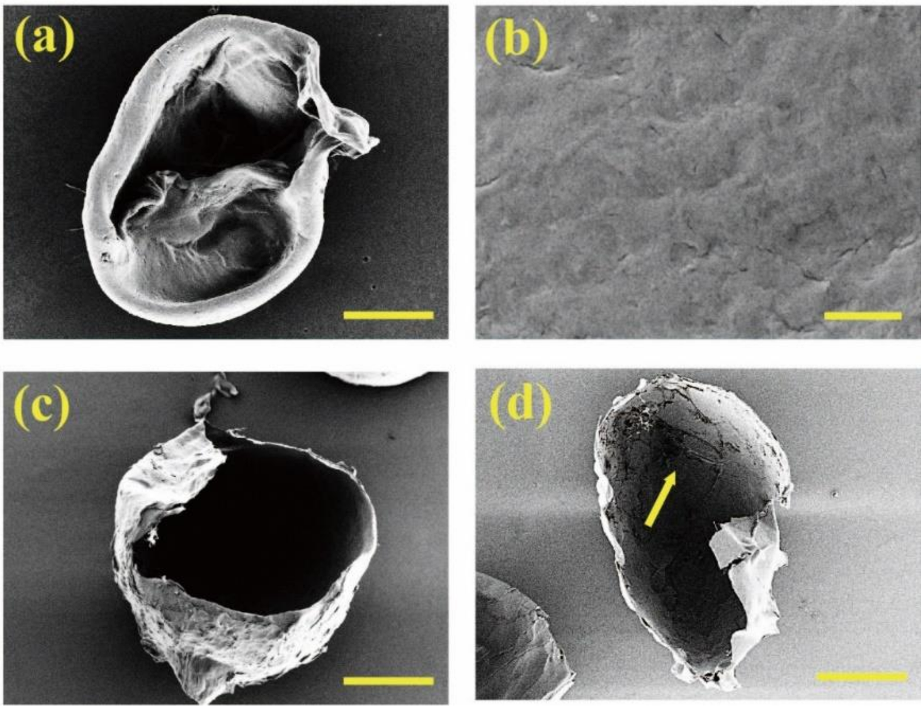

Fig. S2 SEM images of core-shell microcapsules. **(a)** Collapse of ALG microcapsules without CNC. **(b)** The surface of ALG microcapsules without CNC. **(c)** Microcapsules without stem cells. **(d)** Microcapsules with stem cells. Scale bar in **(a, c and d)** is 100 μm and in **(b)** is 10 μm

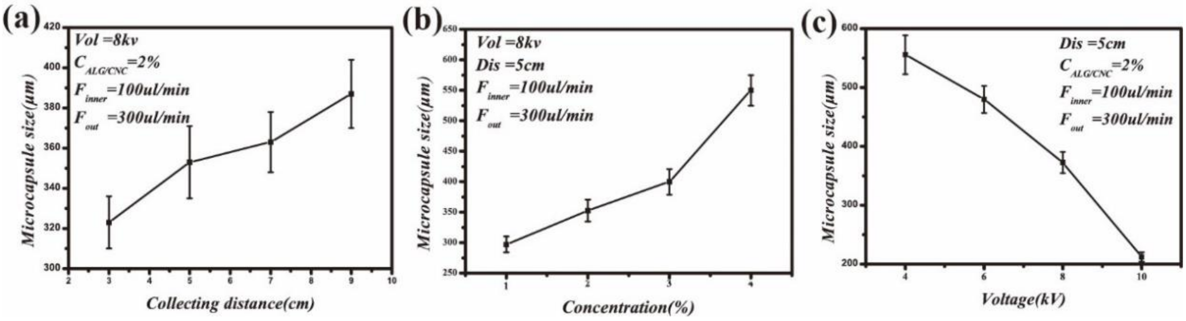

Fig. S3 Microcapsules with different diameters. The influences of **(a)** collection distances, **(b)** concentrations and **(c)** voltage on microcapsules diameter

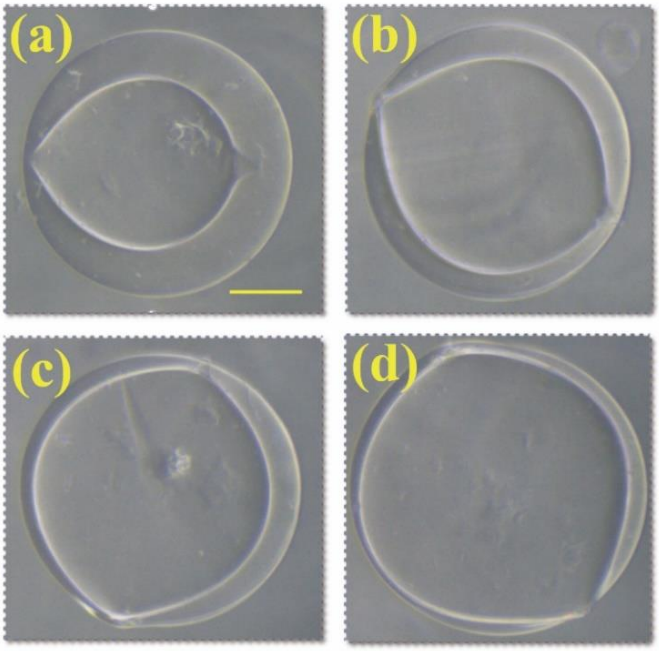

Fig. S4 Microcapsules with different diameters and morphologies were obtained by adjusting the flow rate of internal phase (a-d: 50, 100, 150, 200 μL/min, respectively). Voltage = 8 kv, collection distances = 5 cm, concentration = 2% and the flow rate of outer phase = 300 μL/min, respectively. Scale bar is 100 μm.

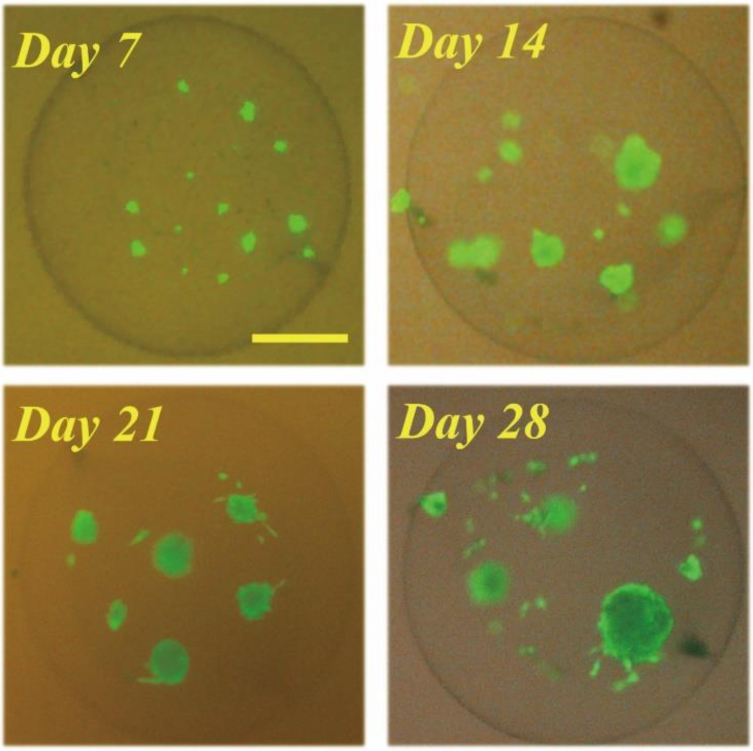

Fig. S5 The morphology and proliferation conditions of cells cultured in the microcapsules for 7-28 days. Scale bar is 100 μm

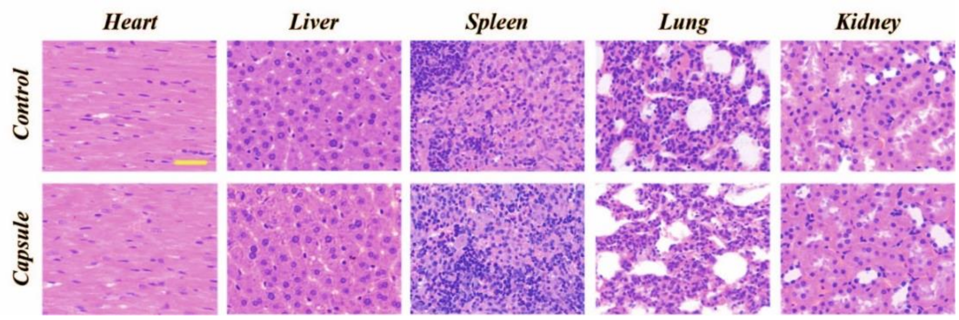

Fig. S6 HE stained main organs in Control and Capsule groups. Scale bar is 100 μm

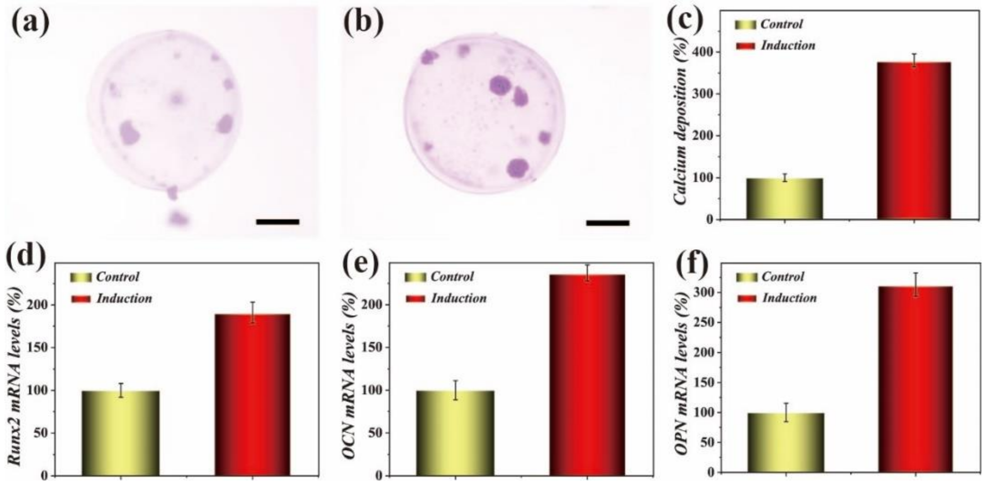

Fig. S7 Osteogenic potential of BMSCs in control and osteogenic induction microcapsule on 21 days. **(a, b)** Alizarin red-stained microspheres in **(a)** control and **(b)** osteogenic induction group. **(c)** Relative percentage calcium deposition. (d, e and f) Relative mRNA expression of osteogenesis-related genes, including Runx2, Ocn, Opn. Scale bar is 100 μm

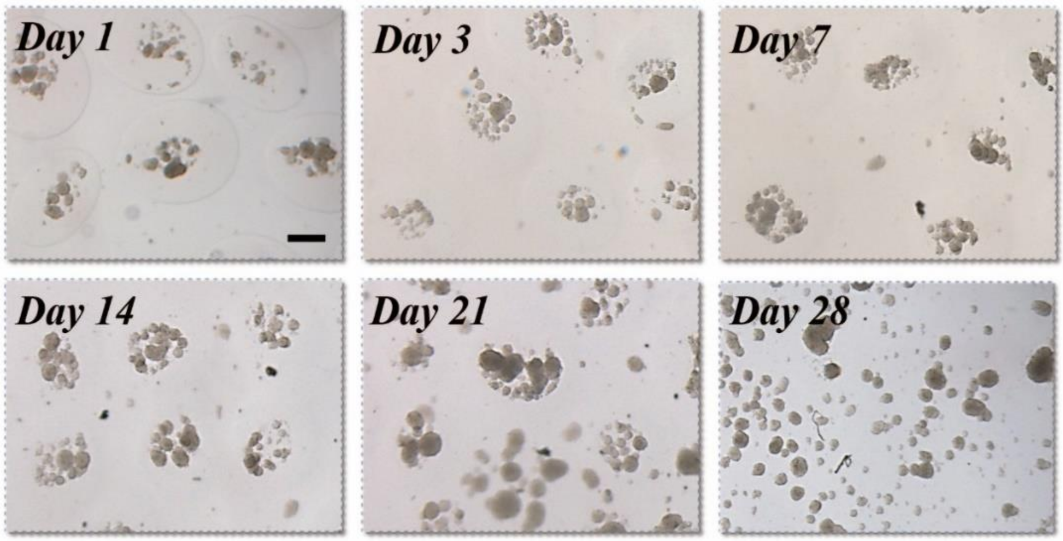

Fig. S8 BMSC cluster release from the microcapsules over 4 weeks *in vitro*. Scale bar is 100 μm

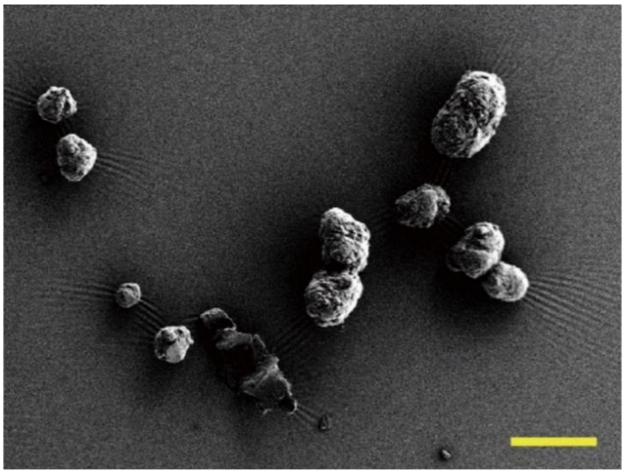

Fig. S9 SEM of BMSC cluster released from the microcapsules. Scale bar is 100 μm

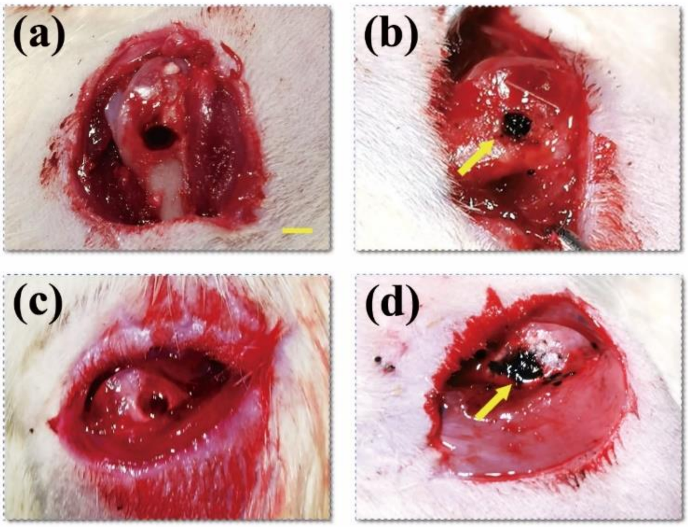

Fig. S10 The labeled microcapsules after treatment. **(a)** The control bone defect model on 0 day. **(b)** The labeled microcapsules treated bone defect on 0 day. **(c)** The control bone defect model after treatment for two weeks. **(d)** The labeled microcapsules in bone defect site after treatment for two weeks. The yellow arrow indicates the labeled microcapsules. Scale bar is 2 mm.

Table S1 Primer sequences used for RT-Qpcr

Gene	Forward primer sequence (5′-3′)	Reverse primer sequence (5′-3′)	Tm (°C)
Runx2	AGACCAGCAGCACTCCAT AT	CTCATCCATTCTGCCGCTAGA	60
Ocn	GGTGGTGAATAGACTCCGGC	GCAACACATGCCCTAAACGG	60
Opn	GAGGAGAAGGCGCATTACAG	ACAGAATCCTCGCTCTCTGC	60
Gapdh	GGCACAGTCAAGGCTGAGAATG	ATGGTGGTGAAGACGCCAGTA	60
